# Exploring the strengths and limitations of AI-driven variant prioritization versus manual curation in inborn errors of immunity

**DOI:** 10.3389/fgene.2026.1713299

**Published:** 2026-03-04

**Authors:** Laith Ibrahim Moushib, Nerea Moreno-Ruiz, Andrea Martín-Nalda, Jacques G. Rivière, Blanca Urban, Romina Dieli-Crimi, Janire Perurena-Prieto, Aina Aguiló-Cucurull, Elena Pérez-Estévez, Xavier Solanich, Pere Soler-Palacín, Roger Colobran, Laura Batlle-Masó

**Affiliations:** 1 Translational Immunology Research Group, Vall d’Hebron Research Institute (VHIR), Vall d’Hebron Barcelona Hospital Campus, Barcelona, Spain; 2 Jeffrey Modell Diagnostic and Research Center for Primary Immunodeficiencies, Barcelona, Spain; 3 Department of Cell Biology, Physiology and Immunology, Autonomous University of Barcelona (UAB), Bellaterra, Spain; 4 National Center of Hematology, Al-Mustansiriyah University, Baghdad, Iraq; 5 Department of Medicine and Life Sciences, Institute of Evolutionary Biology (UPF-CSIC), Universitat Pompeu Fabra, Barcelona, Spain; 6 Infection and Immunity in Pediatric Patient, Vall d’Hebron, Research Institute (VHIR), Vall d’Hebron Barcelona Hospital Campus, Barcelona, Spain; 7 Pediatric Infectious Diseases and Immunodeficiencies Unit, Hospital Universitari Vall d’Hebron (HUVH), Vall d’Hebron Barcelona Hospital Campus, Barcelona, Spain; 8 Immunology Division, Hospital Universitari Vall d’Hebron (HUVH), Vall d’Hebron Barcelona Hospital Campus, Barcelona, Spain; 9 Pediatric Intensive Care Unit (PICU), Cruces University Hospital, Bilbao, Spain; 10 Primary Immunodeficiency Unit, Internal Medicine Department, Hospital Universitari de Bellvitge, L’Hospitalet de Llobregat, Spain; 11 Clinical Sciences Department, Faculty of Medicine and Health Sciences, University of Barcelona, Barcelona, Spain; 12 Department of Clinical and Molecular Genetics, Hospital Universitari Vall d’Hebron (HUVH), Vall d’Hebron Barcelona Hospital Campus, Barcelona, Spain

**Keywords:** artificial intelligence, genetic diagnosis, inborn errors of immunity, variant prioritization, whole genome sequencing

## Abstract

**Introduction:**

Next-generation sequencing (NGS) has transformed the genetic diagnosis of human diseases, yet many patients remain unsolved due to the complexity of variant interpretation. Manual curation of candidate variants is effective but time-consuming and requires specialized expertise. Artificial intelligence (AI)-driven platforms have emerged as scalable tools for variant prioritization, yet their performance compared with manual curation remains insufficiently evaluated. The aim of this study was to evaluate the performance of AI-driven platforms for variant prioritization in a cohort of patients with inborn errors of immunity (IEI) and to compare their strengths and limitations with manual curation.

**Methods:**

We analyzed 22 unsolved IEI cases that had previously undergone inconclusive NGS studies. Whole-genome sequencing was performed, and variant prioritization was carried out using two AI-driven platforms -AIMARRVEL and AION (Nostos Genomics)- and by manual curation. Selected variants were classified according to clinical relevance (very high, high, medium, or low), integrating both molecular and phenotypic evidence.

**Results:**

Across the cohort, AI platforms efficiently prioritized variants with clear pathogenic features, often reaching the same conclusions as manual curation but in a fraction of the time. One patient (5%) received a conclusive diagnosis (*FAM111B*), and four patients (18%) carried variants of high clinical relevance, including strong disease-causing candidates in *CD247* and *SH2B3*. Additional medium-relevance variants were identified in 36% of cases, although evidence was insufficient for functional validation. Notably, concordance between AIMARRVEL and AION was limited, particularly for variants of uncertain significance (VUS), reflecting differences in algorithmic weighting of variant features versus clinical phenotype. Both platforms also highlighted potentially novel associations in *RUNX1* and *TRAF7*, underscoring their capacity to extend beyond classical IEI genes.

**Discussion:**

Our results show that AI-driven tools are powerful for detecting clearly pathogenic variants and can markedly accelerate the diagnostic process. However, their strong reliance on curated databases, limited incorporation of phenotypic data, and challenges in handling VUS may reduce their effectiveness. Enhancing phenotype integration, expanding annotations (including non-coding regions), and incorporating up-to-date literature could improve their performance. Ultimately, AI tools should complement expert curation, with future models evolving toward integrative approaches that better capture the complexity of human disorders.

## 1 Introduction

Advancements in genomic technologies have been crucial in improving rare disease diagnostics, particularly by enabling earlier and more accurate identification of pathogenic mutations. As a result, clinicians and researchers now utilize a broad range of genomic approaches, including targeted gene panels, whole exome (WES) and whole genome (WGS) sequencing with both short- and long-read methods, as well as transcriptomics and epigenetic technologies. In the field of inborn errors of immunity (IEIs), these advancements have significantly contributed to identifying novel disorders and expanding our understanding of their genetic causes (Vorsteveld et al., 2021; von Hardenberg et al., 2024), as exemplified by the latest update of the International Union of Immunological Societies (IUIS) listing over 500 genes associated with inborn errors of immunity (Poli et al., 2025). This number continues to rise with ongoing developments in genomic technologies and computational tools.

Identifying genetic variants linked to IEI or any other rare Mendelian disease has traditionally been a complex and time-consuming process. Although data processing and quality assessment is mainly automated, manual curation needs to be performed to identify a subset of genetic variants likely to be causing the clinical phenotype. This curation is labor-intensive and requires expertise that can lead to different interpretations of the findings. To standardize the interpretation of genetic variants, the American College of Medical Genetics and Genomics (ACMG) guidelines are commonly used (Richards et al., 2015), classifying the variants in five categories regarding their potential effect: pathogenic, likely pathogenic, variant of uncertain significance (VUS), likely benign and benign.

In complex IEI cases where no diagnostic variants (pathogenic or likely pathogenic) are identified, the focus shifts toward the interpretation of VUS. While definitively determining the clinical impact (pathogenicity) of these VUS ultimately requires functional assays, a meticulous preliminary assessment to prioritize the most promising VUS candidates is an essential first step (von Hardenberg et al., 2024; Spielmann and Kircher, 2022). Particular attention is given to rare or novel VUS in relevant IEI genes that display notable genomic features suggestive of potential pathogenicity, such as high evolutionary conservation scores and location within exonic hotspots harboring pathogenic variants. Prioritizing these candidates for further analysis is a labor-intensive process involving comprehensive literature review, database queries, and computational predictions. The task becomes even more challenging when interesting variants are detected in genes not previously associated with IEI, as establishing their direct immune involvement demands rigorous additional steps such as verification of protein expression in relevant immune cells and elucidating protein-protein interactions with potential roles in immune function (Khan and Butte, 2021).

Despite all these efforts and advances, establishing a genetic diagnosis in IEI patients remains difficult, with approximately 60%–75% of cases remaining undiagnosed regardless of the genomic approach used (Vorsteveld et al., 2021; Engelbrecht et al., 2021; Okano et al., 2020; Rudilla et al., 2019). This is due to many factors, including the clinical heterogeneity of IEI patients, a wide range of symptoms that can overlap with other conditions, the complex interplay of genetic factors (e.g., epistatic effect, modifier genes), atypical/unconventional inheritance patterns, the diversity of the genetic mechanisms underlying IEI (loss-of-function, dominant-negative interference, hypomorphic effects, or gain-of-function) and incomplete knowledge of genes involved in immune function (Akalu and Bogunovic, 2024).

With that, the interpretation of VUS continues to pose a significant diagnostic challenge and holds the key to future advances in the field. Accurate assessment of these variants could help prioritize both rare and phenotype-relevant variants in IEI genes, as well as novel candidate genes. The identification of novel genes is complicated by the complex filtering of the large number of variants and the limited annotations of some of them. Therefore, new tools guiding clinicians and researchers toward the most promising targets for further investigation in challenging IEI cases are needed to contribute to the advance of the field.

The integration of artificial intelligence (AI) tools into variant prioritization for rare disease genomics is progressing rapidly, offering new possibilities for faster and more accurate genetic diagnoses (O’Brien et al., 2022; De La Vega et al., 2021; Huang et al., 2022). Unlike manual curation, which relies on sequential application of defined filtering criteria, AI models can dynamically synthesize and weight multiple lines of evidence across vast genomic datasets, with the ability to analyze over a hundred variant-level features simultaneously (Abdelwahab and Torkamaneh, 2025). This capacity enables AI systems to uncover complex genetic patterns and novel gene-disease associations that may be missed by traditional methods (manual curation), proving invaluable for otherwise unsolved cases.

In this study, we aimed to evaluate two AI-driven variant prioritization tools–AIMARRVEL (freely available software) (Mao et al., 2024) and AION from Nostos Genomics–versus traditional manual curation in unsolved IEI cases. These cases had previously undergone genetic testing (either gene panels or whole exome sequencing) without yielding a genetic diagnosis. We performed short read WGS and designed a comparative assessment between manual and AI-driven variant prioritization. We evaluated the ability of AI tools to identify genetic variants causing the clinical phenotype of the studied cases; the capacity of AI tools to prioritize additional rare, clinically relevant variants in other IEI genes; and the potential of the AI tools to identify variants in genes not present within the known IEI candidate genes but potentially relevant to the patient’s phenotype (novelty assessment). With that, we discuss the benefits and limitations of AI-driven approaches for variant prioritization and demonstrate their utility in the rare disease context.

## 2 Materials and methods

### 2.1 Patient cohort

This study included 22 patients (referred to by C1 to C22) presenting clinical features suggestive of underlying genetic defects in immune-related genes. Only patients with a previous inconclusive genetic study (IEI gene panel or WES) were included. Clinical data, including detailed assessments and laboratory assays, were collected for all participants. Clinical manifestations were systematically documented using Human Phenotype Ontology terms (HPO) (Gargano et al., 2024) (Supplementary Table S1). This was done by the referring clinician of each patient, based on the clinical records and the available lab results. Our cohort includes 12 males and 10 females, all with Central European origin (mainly Spanish), except for two probands of North-African origin and one from South America. Three patients have a history of consanguinity: in two cases, the parents were first cousins, and in the third case, the paternal grandparents were cousins. Two probands died at an early age, among the others, there are 8 pediatric patients and 12 adults, with a median age of 21 years old (average 20 years old).

Out of the 22 cases, genomic DNA from the proband and both biological parents was extracted in 19 cases. For the remaining 3 cases: in C13 only the proband and the mother were available, in C9 the proband, the father and an affected twin were available; and in C22 the proband, both parents and an affected sibling were available, leading to a total of 66 samples. Only index cases were used for the main analysis, while familial data were used for segregation analysis of the shortlisted candidate variants (Supplementary Figure S1).

### 2.2 Whole genome sequencing

Peripheral blood for all cases and selected family members was collected in EDTA tubes (BD, USA) for DNA extraction. Genomic DNA libraries were prepared using the TruSeq DNA PCR-Free Kit (Illumina, San Diego, CA, USA) and sequenced on an Illumina NovaSeq 6000 System (150-bp paired-end reads). Sequencing quality was assessed for all samples prior to analysis. Genome sequencing data was processed using a standard pipeline, including read alignment to the human reference genome (GRCh37/hg19) with BWA-MEM v0.7.17 (Li and Durbin, 2009), duplicate marking with Picard v2.18.2 (https://broadinstitute.github.io/picard/), and variant calling using GATK v4.0.5.1 (McKenna et al., 2010).

### 2.3 Study design and comparative assessment of AI and manual approaches for variant prioritization

While a growing number of AI-based variant prioritization tools are currently available, we deliberately focused on web-based platforms suitable for clinical environments that do not require in-house bioinformatics expertise, which is often limited in routine clinical practice. Based on these criteria, and taking into account resource considerations, we selected the platforms AIMARRVEL (hereafter referred to as AIM) and AION from Nostos Genomics (hereafter referred to as AION).

Same VCF files and HPO terms were used for each case as input in both AI-driven platforms.

In AIM, variants are prioritized using the AIMARRVEL pathogenicity score, with higher scores indicating greater likelihood of disease relevance. Results are categorized as “Default,” “Recessive,” “ND” (novel disease), and “ND recessive”. For the main comparative analysis, the top 10 variants from AIM’s “Default” category -those assigned the highest prediction scores- were selected as the study dataset. From there, we filtered out variants seen in more than three patients and genes with high number of variants, leading to a final dataset of 211 variants including all probands and the two affected siblings (Supplementary Table S2).

Similarly, AION assigns each variant an AION Score that ranges from 0% (benign) to 100% (pathogenic), with VUS and “No Evaluation” also being assigned. AION outputs variants in two main categories: “Smoking Guns” (high-confidence candidates) and “AION Clues” (variants with weaker or incomplete evidence). All prioritized variants from both categories were included in the analysis for each case, excluding common variants and highly polymorphic genes, we kept a dataset of 220 variants (Supplementary Table S3).

For both tools we first analyzed “Strong Candidates” including variants with an AIM score equal or higher to 0.1 and variants marked as Smoking Guns in AION. Next, we selected variants present in the genes associated with inborn errors of immunity as defined by the IUIS (Poli et al., 2025) (Supplementary Table S4). We reported the candidate variants in tabular format, including the most important annotations for each variant and a column with the clinical relevance classification (low, medium, high and very high) based on characteristics of the gene and variant, and their relation to the clinical phenotype. This classification was essential to convey overall clinical relevance, as it integrates contextual and expert-driven information (such as phenotype concordance and biological plausibility) that cannot be easily or directly captured by automated metrics alone.

### 2.4 Manual curation: variant filtering and prioritization

For manual curation, the initial common dataset of VCF files was annotated using SnpEff, incorporating additional databases such as ClinVar and dbNSFP. Pathogenicity predictors including CADD (GRCh37-v1.6) (Rentzsch et al., 2019) and REVEL (Ioannidis et al., 2016) were used to assess variant impact. Each variant was also flagged if present in the IUIS list candidate genes (Supplementary Table S4).

Variants were filtered using the following thresholds: genotype quality (GQ) > 20, allele balance inside 0.2–0.8, read depth >10, and variant quality score (QUAL) > 500. Population allele frequency thresholds were applied using gnomAD (v4.1.0; https://gnomad.broadinstitute.org/), retaining only rare (MAF <0.05) or novel variants. Variants with high or moderate predicted impact, especially those affecting coding regions or canonical splice sites, were prioritized. All shortlisted variants were reviewed by clinical geneticists, integrating ACMG guidelines, clinical phenotype, and segregation data to support final interpretation. When relevant, a literature review querying variant interpretation databases such as Franklin (https://franklin.genoox.com/clinical-db/home), Varsome (https://varsome.com/), and ClinVar (https://www.ncbi.nlm.nih.gov/clinvar) was also conducted.

Clinically relevant variants were defined as rare or novel variants occurring in genes with phenotypic overlap with patient symptoms (as assessed by HPO terms). To ensure fair comparison with AI approaches, familial genetic data was only used for annotation and not for hard filtering, as this feature could not be included in both AI approaches. For cases C9 and C22, in which affected siblings were also sequenced (C9s and C22s respectively), only shared variants were considered during manual curation.

## 3 Results and discussion

### 3.1 Evaluating concordance and discrepancies between AI platforms in prioritizing phenotype-relevant variants

**FIGURE 1:**
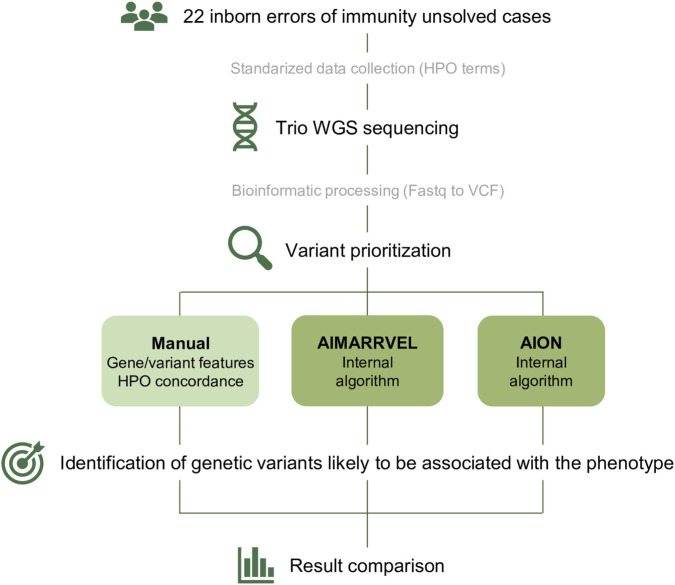
Overview of the used workflow. Twenty-two unsolved IEI cases were selected and clinical data was collected and codified using HPO terms. Whole genome sequencing (WGS) was performed to the index cases and both parents and affected siblings when present. Bioinformatic processing of the dataset was done to generate VCF files. HPO terms and VCF files were used to perform three variant prioritization strategies: Manual curation; AIMARRVEL and AION. Dark green indicates AI based approaches.

Our study included 22 IEI cases in which WGS was performed on probands, their parents, and any affected siblings (Supplementary Figure S1). All patients had previously undergone genetic testing using either an IEI-targeted gene panel (461 genes) or WES, of which yielded negative results (i.e., no pathogenic variants were identified). Sequencing output and clinical data were standardized and analyzed for variant prioritization using a manual approach and two AI-based platforms: AIM and AION (Figure 1).

We started by analyzing variants prioritized as strong candidates by both platforms, including AIM top picks (AIM score >0.1) and AION Smoking Guns. A total of 14 variants were selected using these filters (with the number of strong candidates per patient ranging from zero to two): 11 by AIM, two by AION, and 1 by both approaches (Table 1). All but one, were classified as pathogenic or likely pathogenic according to ACMG guidelines. All variants exhibited extremely low population frequencies and high evolutionary conservation scores. Through expert manual curation, these variants were assessed for their clinical relevance (Table 1, last column). The aim of this classification was to evaluate each variant in the context of the patient’s clinical phenotype, in order to guide subsequent steps for validation and potential clinical management. We identified three clinically relevant variants: one classified as being of very high relevance (in *FAM111B*) and two as of high relevance (in *G6PD* and *BRCA1*).

**TABLE 1:** Genetic variants classified as strong candidates (AIM score >0.1 and/or AION Smoking Guns) detected by AI approaches.

Method	Case	Gene_HGNC	OMIM	DNA_HGVS	AA_HGVS	Effect	Zygosity	Segregation	gnomAD_v4.1	CADD_v1.6	ACMG	Clinical relevance
AIM	C2	*ALG14*	612866; AR	c.310C>T	p.Arg104Ter	Stopgain	Het	Maternal	8.74E-05	38	P	Low
AIM	C5	*SLC19A2*	603941; AR	c.1063A>C	p.Lys355Gln	Missense	Het	Paternal	3.84E-05	24.1	LP	Low
AIM	C8	*SLC25A11*	604165; AD	c.673T>A	p.Cys225Ser	Missense	Het	Paternal	3.10E-06	23.7	LP	Low
AION	C8	*BRCA1*	113705; AD, AR	c.845C>A	p.Ser282Ter	Stopgain	Het	Paternal	.	35	P	High
AIM	C10	*CTNS*	606272; AR	c.922G>A	p.Gly308Arg	Missense	Het	Maternal	2.98E-05	32	P	Low
AION, man^a^	C10	*G6PD*	305900; XL	c.844G>C	p.Asp282His	Missense	Hemi	Maternal	8.92E-04	24.4	P	High
AIM	C12	*GCDH*	608801; AR	c.263G>A	p.Arg88His	Missense	Het	Paternal	.	32	P	Low
AIM	C12	*ACADS*	606885; AR	c.417G>C	p.Trp139Cys	Missense	Het	Maternal	2.42E-05	29.7	P	Low
AIM	C13	*XPA*	611153; AR	c.436C>T	p.Gln146Ter	Stopgain	Het	Maternal	.	37	P	Low
AIM	C14	*STRA6*	610745; AR	c.961A>C	p.Thr321Pro	Missense	Het	Maternal	7.29E-04	23.3	VUS	Low
AIM	C16	*TMEM67*	609884; AR	c.1046T>C	p.Leu349Ser	Missense	Het	Maternal	4.11E-05	27.9	P	Low
AIM	C19	*MLH1*	120436; AR; AD	c.1667 + 1G>A	.	Splicing	Het	Maternal	.	28.7	P	Low
AION, AIM	C20	*FAM111B*	615584; AD	c.1883G>A	p.Ser628Asn	Missense	Het	De novo	.	22.8	P	Very high
AIM	C22	*AGXT*	604285; AR	c.614C>T	p.Ser205Leu	Missense	Het^b^	Maternal	.	24.2	P	Low

^a^ Also found in AIM, but not highlighted as strong candidate (AIM, score 0.03).

^b^ Also heterozygous in the affected sibling.

The *FAM111B* p. Ser628Asn heterozygous variant was identified by both AIM and AION in patient C20. This variant was absent from population reference databases (gnomAD), occurred at a highly conserved position (CADD: 22.8), and was confirmed to be *de novo*. The p. Ser628Asn variant had previously been reported as pathogenic, associated with hereditary fibrosing poikiloderma with tendon contracture, myopathy, and pulmonary fibrosis (POIKTMP, OMIM #615704) (Mercier et al., 2013). The patient was initially evaluated for a possible IEI due to the presence of eczematoid dermatitis, ectodermal dysplasia, eosinophilia, respiratory insufficiency, and recurrent *Staphylococcus aureus* infections. However, the clinical phenotype was ultimately consistent with an atypical presentation of POIKTMP. Based on these findings, the *FAM111B* p. Ser628Asn variant was considered disease-causing in patient C20.

The *G6PD* p. Asp282His hemizygous variant was identified by AION in patient C10. AION classified this variant as a “Smoking Gun,” whereas AIM (Default mode) assigned it a score of 0.03, placing it outside the top-ranked candidates. The variant has a low allele frequency in the reference population (gnomAD: 8.92E-04), and a high conservation score (CADD: 23.5). Pathogenic variants in the *G6PD* gene are associated with congenital non-spherocytic hemolytic anemia-1 (OMIM #300908). The p. Asp282His variant has been reported in multiple populations and is known to reduce enzyme activity to approximately 15% of normal values, consistent with a class III G6PD deficiency (Cappellini et al., 1995). Patient C10 presented with arthritis, *Salmonella* osteomyelitis, abnormalities in memory B- and T-cell subsets, and opportunistic infections. The patient did not have anemia, although red cell testing showed reduced G6PD activity. This finding is consistent with the incomplete penetrance observed in class III variants (Beutler, 2008). Therefore, although this variant accounts for a mild G6PD deficiency (clinically silent in this patient) it cannot explain the full clinical phenotype, although a contributory role cannot be excluded. This variant was also highlighted in the manual filtering approach.

The *BRCA1* p. Ser282Ter heterozygous variant was identified by AION in patient C8. BRCA1 functions as a tumor suppressor, and pathogenic variants are associated with an increased risk of breast and ovarian cancers (Li et al., 2022). In this female patient, the variant was considered a secondary finding; although unrelated to the presenting clinical phenotype, it is clinically significant, and appropriate management was undertaken in accordance with current guidelines (Miller et al., 2021).

**FIGURE 2:**
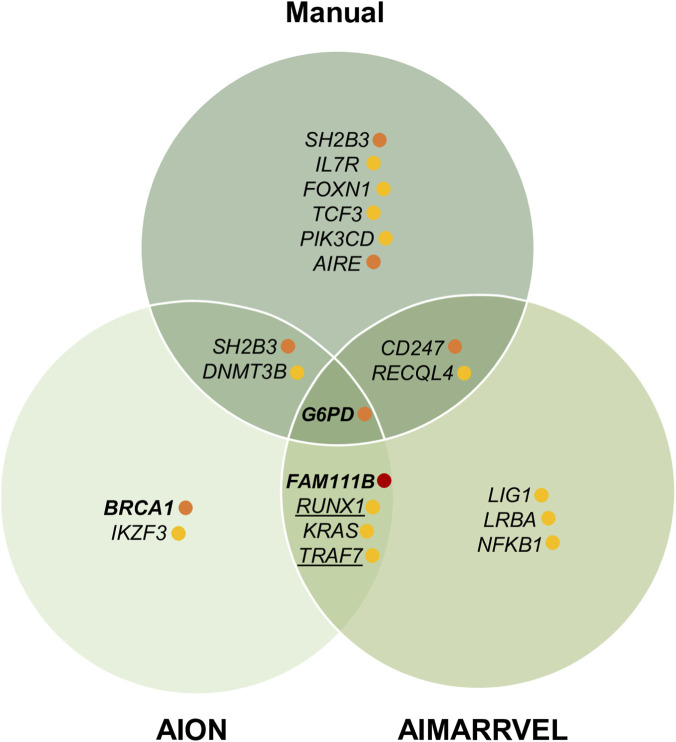
Overlap of prioritized variants identified by manual curation, AIMARRVEL, and AION. Variants with very high clinical impact are indicated with a red dot, those with high impact in orange, and those with medium impact in yellow. Bold indicates strong candidate variants (AIM score >0.1 or AION Smoking Guns); underlining denotes variants in non-IEI genes.

In this initial analysis, where we compared the top-ranked variants selected by both platforms without any manual filtering, we identified 14 variants. Only one variant (p.Ser628Asn in *FAM111B*) was selected by both AION and AIM in the top ranked list (Figure 2). Notably, this variant was the only one classified as both pathogenic and disease-causing, leading to a conclusive genetic diagnosis in the patient. AION identified two additional variants (in *G6PD* and *BRCA1*) that, although not responsible for the patients’ phenotypes, were clinically relevant. The remaining variants, selected exclusively by AIM, were mostly pathogenic or likely pathogenic variants in genes with autosomal recessive inheritance and unrelated to the patients’ phenotypes. These variants likely emerged due to the absence of definitive disease-causing variants in these samples and reflect the random presence of deleterious variants commonly found in the general population (Fridman et al., 2021).

These results underscore the efficiency of AI tools in identifying pathogenic variants. Current AI models typically rely on guideline-based scoring systems and *in silico* predictions, focusing on features such as amino acid changes, evolutionary conservation, and predefined pathogenicity scores. This approach is particularly effective for detecting disease-causing variants and those with significant impacts on protein function, regardless of the patient’s specific phenotype.

However, there is a limited concordance between AI tools which highlights that expert-driven manual curation plays an important role in capturing clinically relevant variants. A key factor underlying this discrepancy is the variation in algorithm design—particularly how features such as predicted molecular impact, population frequency, phenotype similarity, and others are weighted during model training—can contribute to discordant results among automated variant prioritization tools. The influence of each feature within automated approaches has been shown to significantly shape how variants are prioritized (Iancu et al., 2021; Johnson B. et al., 2022; Nicora et al., 2022). For VUS, which lack definitive evidence for pathogenicity or benignity, prioritization may be more sensitive to differences in algorithmic feature weighting and threshold settings. This can lead to variable outcomes, especially in models that rely heavily on internally generated pathogenicity scores (e.g., AIM and AION’s variant scoring systems).

### 3.2 Assessment of variant prioritization by AI platforms and manual curation in IEI genes

In the previous analysis, one out of 22 patients was considered “solved,” as both AION and AIM identified a disease-causing variant that was concordant with the phenotype after manual curation. Next, we focused on the remaining 21 patients, specifically analyzing variants in IEI-associated genes, which are the most likely candidates to explain the phenotype. Although most–but not all–of these genes had been previously examined through genetic tests (gene panels or WES) without conclusive results, we aimed to assess variant prioritization by AION and AIM, compared with our manual curation. While we did not expect to identify clearly disease-causing variants (since such variants should have appeared in the initial analyses), we sought to determine whether any genetic variants merited further investigation regarding their potential role in the patients’ phenotypes. In this dataset, as in the previous one, we assessed the clinical relevance of the candidate variants and gave them a classification of low, medium or high based on the relevance of each variant in the context of the patient’s clinical phenotype. We identified 15 variants (Table 2), four of them with a high score.

**TABLE 2:** Genetic variants in IEI genes detected by AI approaches and by manual curation with a relevant clinical significance.

Method	Case	Gene_HGNC	OMIM	DNA_HGVS	AA_HGVS	Effect	Zygosity	Segregation	gnomAD_v4.1	CADD_v1.6	ACMG	Clinical relevance
Man	C3	*SH2B3*	187950; na	c.149G>T	p.Arg50Leu	Missense	Het	Maternal	9.12E-06	22.4	VUS	High
AION, man	C3	*SH2B3*	605093; na	c.1193G>T	p.Arg398Leu	Missense	Het^a^	Paternal	1.24E-06	28.4	VUS	High
Man	C9	*IL7R*	146661; AR	c.419G>A	p.Arg140Gln	Missense	Hom^b^	Paternal/NA	3.54E-04	21.5	VUS	Medium
AIM, man	C10	*CD247*	186780; AR^c^	c.301C>T	p.Gln101Ter^c^	Stopgain	Het	Paternal	2.73E-04	46	P	High
AION	C12	*IKZF3*	606221; AD	c.1289C>A	p.Pro430His	Missense	Het	Paternal	.	26.2	VUS	Medium
AIM	C13	*LIG1*	126391; AR	c.628del	p.Glu210AsnfsTer34	Frameshift	Het	Maternal	.	.	LP	Medium
Man	C13	*FOXN1*	600838; AD, AR	c.56T>C	p.Leu19Pro	Missense	Het	NA	3.90E-04	26.2	VUS	Medium
AIM	C16	*LRBA*	614700; AR	c.6488_6489 delAG_insCT	p.Lys2163Thr^d^	Missense	Het	Maternal	.	24.7	VUS	Medium
AIM, man	C17	*RECQL4*	603780; AR	c.2263C>T	p.Arg755Trp	Missense	Hom	Paternal/maternal	1.86E-06	33	VUS	Medium
Man	C17	*TCF3*	147141; AD, AR	c.16A>G	p.Arg6Gly	Missense	Het	Paternal	9.56E-05	25.4	VUS	Medium
AION, AIM	C19	*KRAS*	190070; AD	c.88G>A	p.Asp30Asn	Missense	Het	Maternal	.	29.3	VUS	Medium
AIM	C20	*NFKB1*	164011; AD	c.2281T>A	p.Trp761Arg	Missense	Het	Maternal	6.57E-05	21.9	VUS	Medium
Man	C20	*PIK3CD*	602839; AD, AR	c.1921C>T	p.Arg641Cys	Missense	Het	Maternal	9.30E-06	25	VUS	Medium
AION, man	C21	*DNMT3B*	602900; AD	c.2254G>A	p.Asp752Asn	Missense	Het	Maternal	.	28.8	VUS	Medium
Man	C22	*AIRE*	607358; AD, AR	c.901G>A	p.Val301Met	Missense	Het^b^	Paternal	8.15E-04	25	VUS	High

^a^ Heterozygous in the unaffected sibling.

^b^ Heterozygous in the affected sibling.

^c^ A recent study demonstrated that the variant p. Gln101Ter acts through a dominant-negative mechanism (autosomal dominant) (Briones et al., 2024).

^d^ This variant was initially reported by the software as two consecutive independent variants. Manual inspection with IGV, confirmed that both changes were in cis and represented a single indel event.

Patient C3 carried two very rare heterozygous missense variants in *SH2B3* (p.Arg50Leu and p. Arg398Leu), each inherited from a different parent (Table 2). AION prioritized only the p. Arg398Leu variant, while both variants were selected after our manual curation. Clinically, patient C3 presented with a complex autoimmune phenotype, including hypothyroidism, anemia, diarrhea, recurrent infections, urticaria, and malar rash. Although *SH2B3* is not a classical IEI gene, it was recently included in the IUIS 2024 classification as causative of a novel autosomal recessive syndrome characterized by myeloproliferation and multiorgan autoimmunity, with remarkable phenotypic heterogeneity across reported patients (Poli et al., 2025; Blombery et al., 2023; Perez-Garcia et al., 2013; Leardini et al., 2025). The high CADD scores (21.5 and 31), together with the compound heterozygous state and partial phenotypic concordance, prompted us to further investigate the functional impact of the *SH2B3* variants (experiments ongoing). Notably, AIM did not prioritize either variant, underscoring a limitation of its current model: the tendency to under-recognize rare, phenotype-relevant VUS, particularly when they are insufficiently represented in training datasets or scoring frameworks. In contrast, AION shortlisted p. Arg398Leu based on a high internal pathogenicity score, but deprioritized p. Arg50Leu by classifying it as benign, despite its ACMG designation as a VUS. Furthermore, AION failed to capture the gene-disease association because it relies on OMIM, which currently only documents somatic *SH2B3* variants associated with myeloproliferative neoplasms (OMIM *605093). The germline biallelic *SH2B3* syndrome included in the latest IUIS classification (Poli et al., 2025) has not yet been incorporated into OMIM. The fact that *SH2B3*-related diseases in OMIM are exclusively based on somatic variants may have influenced AION not to prioritize a second very rare variant in a gene where one such variant had already been selected. This highlights a broader limitation of AI-driven platforms when dependent on external resources such as OMIM that may lag behind the most recent genetic discoveries.

In patient C10, we had previously identified a *G6PD* variant consistent with mild G6PD deficiency, although this did not fully explain the patient’s clinical phenotype (see Table 1 and previous results section). In the present IEI-focused analysis, AIM prioritized a stop-gain variant in *CD247* (p.Gln101Ter), inherited from the father (Table 2). This variant was also selected by our manual curation. *CD247* encodes the CD3 zeta chain of the T-cell receptor, which is essential for coupling antigen recognition to downstream intracellular signaling pathways. *CD247* deficiency typically results in a form of severe combined immunodeficiency with autosomal recessive inheritance (Rieux-Laucat et al., 2006). However, recent work by J.R. Regueiro’s group demonstrated that heterozygous stopgain *CD247* variants expressed at the cell surface (encoding a truncated CD3 zeta chain that retains the transmembrane region) exert a dominant-negative effect, leading to immunodeficiency with incomplete penetrance (Briones et al., 2024). Remarkably, in that study, four individuals were reported to carry the heterozygous *CD247* p. Gln101Ter variant: three presented with T-cell abnormalities and infections or autoimmunity, whereas one remained asymptomatic. Notably, the clinical symptoms in affected individuals improved with age. Given the T-cell dysfunction and infectious profile observed in patient C10, the *CD247* p. Gln101Ter variant emerges as a strong candidate for the molecular cause of the disease in this case.

In patient C22, neither AIM nor AION prioritized any variant. However, manual curation revealed a heterozygous missense variant in *AIRE* (c.901G>A; p. Val301Met), previously reported to cause an autosomal dominant form of autoimmune polyendocrine syndrome type 1 (APS-1) through a dominant-negative mechanism, with incomplete penetrance and typically later onset (Oftedal et al., 2015; Oftedal et al., 2023). C22 presented with profound T-cell lymphopenia, detected early through the SCID newborn screening program (Argudo-Ramírez et al., 2019). Interestingly, her older brother also showed T-cell lymphopenia on newborn screening and was found to carry the same variant. T-cell lymphopenia has not been described in either the recessive or the dominant forms of APS-1, making the contribution of the *AIRE* p. Val301Met variant to the patient’s phenotype uncertain. Notably, the variant was inherited from the father, who displayed clinical features consistent with the monoallelic form of APS-1: vitiligo, alopecia areata and onychomycosis. Two main factors may explain why neither AIM nor AION prioritized this variant: (1) despite published evidence (Oftedal et al., 2015; Oftedal et al., 2023), its classification remains controversial, being more frequently reported as a VUS than as likely pathogenic or pathogenic, potentially due to its population frequency (MAF = 0.0008) and associated incomplete penetrance; and (2) the lack of genotype–phenotype correlation in the two affected siblings.

The remaining variants in Table 2 were scored as medium, indicating that while we do not consider them disease-causing, we cannot exclude the possibility that some may contribute to the patients’ clinical phenotypes.

Overall, this IEI-targeted analysis revealed limited concordance between the variants prioritized by AIM and AION (Figure 2). Only one variant was selected by both platforms (p.Asp30Asn in *KRAS*) which we initially considered of interest given its absence in population databases and its location in the N-terminal region of the gene, where most pathogenic missense variants have been reported (Johnson C. et al., 2022). However, the patient’s phenotype was not consistent with KRAS-associated conditions, and therefore we did not pursue this variant for further validation.

In the absence of a clearly pathogenic variant, this lack of concordance between the AI tools reflects that their prioritization process relies more on variant-related features than on the clinical phenotype. In contrast to AI tools, our manual curation process prioritized rare variants of interest only when there was significant phenotypic overlap with the patient’s presentation, underscoring the central role of phenotype relevance in our prioritization strategy. This highlights a dual challenge for AI-based solutions. First, phenotypic input is provided as HPO terms, and the accuracy and completeness of this annotation directly influences a model’s ability to identify relevant genes and variants (Huang et al., 2022; Zucca et al., 2025). In rare or complex disorders, clinical descriptions are often incomplete or imperfectly mapped to existing HPO terms, despite ongoing improvements (Gargano et al., 2024). As a result, not all features are fully represented, creating gaps in model interpretation. Second, our findings suggest that both AIM and AION ranking strategies rely primarily on molecular impact and existing literature, rather than on phenotype-driven data. In some cases, pathogenic or likely pathogenic variants unrelated to the provided HPO terms were still ranked highly. This indicates that the tools can accurately detect functionally impactful variants but tend to underweight phenotypic relevance. Consequently, clinical terms (here represented by HPO annotations) appear to exert less influence on the algorithms’ decision-making than expected. This may cause clinically important variants to be overlooked, especially in complex diagnostic scenarios. There are other available tools that are more strongly phenotype-driven, such as Exomiser (Vestito et al., 2024), and LIRICAL (Robinson et al., 2020). However, these tools are primarily command-line based and require specialized bioinformatics expertise, placing them outside the scope of the present study.

Additionally, the identification of phenotype-relevant variants may be biased by the composition and size of training datasets. Current AI tools, including AIM and AION, are not trained on IEI-enriched cohorts, which can reduce sensitivity for variants relevant to these disorders (Rivière et al., 2024). Since many IEI cases remain genetically unsolved despite extensive evaluation, expanding and diversifying training datasets to capture the full spectrum of clinically significant variants is essential. Doing so would enhance the ability of AI models to prioritize IEI-relevant variants, particularly in unsolved cases. Finally, current algorithms rely heavily on database-derived information and apply fixed thresholds that limit the integration of new clinical insights or emerging research. While effective for basic filtering, this “static” design hinders performance in complex phenotypes, unsolved cases, or conditions driven by atypical pathogenic mechanisms. As a result, clinically relevant disease-causing variants may be missed.

### 3.3 AI-driven discovery of clinically relevant variants in potentially novel immune-related genes

Having studied the genetic variation in genes linked to IEI, we aimed to extend our analysis to genes not yet associated with these disorders, with the potential to discover new genotype-phenotype associations.

We looked at AIM’s and AION’s top picks (Supplementary Tables S2, S3), looking for genes potentially implicated in immune-related disorders. We prioritized the variants located in genes where emerging literature suggested a role in immune function, and the disruption of which provided a plausible (even partial) explanation for the patient’s clinical presentation. To ensure robustness, we only considered variants identified by both AIM and AION, as we believe that integrating at least two complementary AI tools alongside expert manual review helps mitigate individual tool biases and maximizes the detection of clinically relevant variants that might otherwise be overlooked.

Applying these criteria, both AIM and AION independently prioritized two variants (Table 3). C5 is a patient who presented with gingival overgrowth, acute otitis media, recurrent *Candida* and respiratory infections, colitis and autoimmunity (Supplementary Table S1). Both platforms identified a paternally inherited *RUNX1* variant (c.107C>G; p. Thr36Arg). This variant is extremely rare in the reference population (gnomAD: 1.25E-06) and has a high conservation score (CADD: 28) (Table 3). It is currently classified as a VUS by ACMG, but AION’s scoring system labeled it as pathogenic. *RUNX1* is listed in OMIM (#151385) as disease-causing for familial platelet disorder with associated myeloid malignancy and acute leukemia, both inherited in an autosomal dominant manner. These conditions are not concordant with the phenotype observed in patient C5. However, a comprehensive literature review revealed substantial evidence supporting an important role for *RUNX1* in immune signaling and regulation, which could be relevant to the patient’s clinical manifestations. RUNX1 is a pivotal transcription factor with well-established roles in hematopoiesis, lymphopoiesis, and immune regulation. It influences T cell development and contributes to B cell tolerance and autoimmunity (Thomsen et al., 2021; Wong et al., 2012; Alarcón-Riquelme, 2004; Korinfskaya et al., 2021). Disruption of these functions has been linked to autoimmunity and inflammation, aligning with the manifestations observed in C5 and suggesting that RUNX1 alterations may contribute, at least in part, to the patient’s phenotype.

**TABLE 3:** Genetic variants in genes not previously associated with IEI detected by AI approaches.

Method	Case	Gene_HGNC	OMIM	DNA_HGVS	AA_HGVS	Effect	Zygosity	Segregation	gnomAD_v4.1	CADD_v1.6	ACMG	Clinical relevance
AION, AIM	C5	*RUNX1*	151385; AD	c.107C>G	p.Thr36Arg	Missense	Het	Paternal	1.25E-06	28	VUS	Medium
AION, AIM	C21	*TRAF7*	606692; AD	c.1998 + 1G>A	.	Splice donor	Het	Paternal	1.24E-06	33	VUS	Medium

In patient C21, both AIM and AION prioritized a paternally inherited splicing variant (c.1998 + 1G>A) in *TRAF7*. This variant is extremely rare in the reference population (gnomAD: 1.24E-06), has a high conservation score (CADD: 30) (Table 3), and is predicted to be loss-of-function by altering the splicing process of the gene. While the OMIM entry for *TRAF7* (OMIM #606692) primarily describes cardiac, facial, and digital anomalies with developmental delay (CAFDADD), our patient, apart from mild growth delay, lacked other typical developmental or dysmorphic features. Instead, patient C21 presented with diarrhea, hypogammaglobulinemia, post-vaccination measles infections, and periodic fever (Supplementary Table S1). *TRAF7* encodes a scaffold protein that functions as an E3 ubiquitin ligase, modulating various signaling cascades. Depending on the specific cellular context and stimulus, *TRAF7* can either activate or downregulate NF-κB signaling. For example, it can induce NF-κB downregulation by targeting subunits like NEMO and p65 (RelA) for degradation (Davis and Gack, 2015; Zotti et al., 2011). Recent discoveries have also highlighted an antiviral function for TRAF7 via the activation of antiviral genes and type I IFN responses (Huang et al., 2023). Given the emerging role of *TRAF7* in antiviral immunity, it is plausible to hypothesize that a potential *TRAF7* dysregulation due to the observed aberrant splicing could contribute to the patient’s immune abnormalities, particularly the post-vaccination measles infection.

Although these two variants are unlikely to be directly responsible for the complete phenotype, they may still influence the patients’ clinical presentation. While the patients did not display a phenotype fully compatible with the reported conditions, we identified potential links between the gene functions and certain clinical features (for this reason, we classified them as of medium clinical relevance).

The absence of *RUNX1* and *TRAF7* variants in our manual curation reflects a practical limitation of WGS analysis in clinical settings. Although WGS captures the entire genome, time and resource constraints often preclude truly comprehensive evaluation. Manual review is further challenged by the sheer number and complexity of variants, making interpretation labor-intensive and susceptible to oversight. In contrast, AI-based tools offer a more systematic and scalable alternative. By integrating large-scale datasets and adapting to new knowledge, these platforms can uncover potentially clinically relevant variants that might be overlooked through traditional methods. Our results demonstrate that AI-driven approaches can serve as a valuable complement to manual curation, particularly by broadening the search beyond commonly prioritized genes.

## 4 Conclusion and further directions

In this study, we evaluated genetic variation in 22 cases (C1 – C22) with clinical features suggestive of IEI, all of which had yielded negative results in previous NGS analyses, making them particularly challenging. Manual curation of genetic variants can be highly time-consuming, particularly in cases in which no clear pathogenic variant is readily identified. Accurately quantifying the time required for manual curation compared with AI-based prioritization is inherently challenging, as manual curation time varies widely depending on case complexity, data quality, and the curator’s level of expertise. Consequently, a precise head-to-head comparison of time requirements was beyond the scope of this study. Nevertheless, based on our experience, manual curation of challenging cases such as those included here typically requires approximately 4–8 h per patient, depending on the factors outlined above. AI-driven approaches proved effective for rapid and accurate variant annotation, significantly reducing data analysis time to approximately 1–2 h and enabling implementation in centers without bioinformatics expertise. The only required inputs are a VCF file (typically provided by sequencing facilities) and HPO terms extracted from clinical records. Nevertheless, our analysis also revealed inconsistencies in variant prioritization, particularly in cases lacking a clearly disease-causing variant.

Across the three analyses performed, we identified one variant that we consider unequivocally disease-causing (patient C20, *FAM111B*), accounting for 5% of cases (1/22). In four cases (18%), we detected variants classified as having a high clinical relevance, including strong disease-causing candidates (*CD247* for C10 and *SH2B3* for C3) and others that, while not fully explanatory of the phenotype, remain clinically relevant (*AIRE* for C22 and *BRCA1* for C8) (Figure 2). Eight patients (36%) carried variants classified as being of medium clinical relevance. Although some of these may contribute to the phenotype, current evidence is insufficient to justify further functional characterization. In two patients (C2 and C14, 9%), only variants classified as of low clinical relevance were identified, and in seven patients (32%) no variants of clinical relevance were detected according to our approach.

A limitation of this study is that we restricted the analysis to two AI-driven variant prioritization platforms. Other widely used and well-established tools, such as Exomiser, were not included as benchmarks. Future studies incorporating a broader range of established and emerging variant prioritization approaches will be important to further benchmark AI-assisted methods and to refine best practices for clinical variant interpretation.

Based on our experience with AI-driven platforms (AIM and AION), several important observations and considerations warrant discussion:

1. AI-driven platforms are highly effective at prioritizing clearly disease-causing variants because their algorithms strongly weight features such as predicted loss-of-function consequences, high conservation, and absence from population databases. These variants often align with established pathogenic mechanisms documented in curated resources (e.g., ClinVar, OMIM), making them easier to detect consistently. By rapidly integrating multiple annotation layers, AI tools can highlight such variants with high accuracy, streamlining diagnostic workflows and ensuring that obvious pathogenic candidates are not overlooked, even in centers without extensive bioinformatics expertise. While manual curation can usually reach the same conclusion in identifying causal variants, it is far more time-consuming and resource-intensive. AI platforms therefore provide a faster and more scalable alternative, particularly valuable in routine diagnostics.
2. We observed low concordance between the two AI tools used in this study when prioritizing candidate variants in cases in which no clear pathogenic variant was identified. This finding suggests that combining and comparing outputs from multiple platforms could enhance the robustness of variant prioritization. However, while such a multi-tool approach may be ideal, it is not always feasible in real-world clinical settings due to resource constraints, including cost, computational requirements, and personnel availability. In addition, the proprietary and partially non-transparent nature of some AI platforms, such as AION, may introduce challenges for interpretability and reproducibility, as details regarding the underlying models, feature weighting, and decision-making processes are not fully disclosed to users. This limited transparency may complicate independent validation efforts and the interpretation of discrepancies between different tools, thereby highlighting the continued importance of expert review in both clinical and research settings.
3. A key limitation of current AI-driven platforms is that they often underweight the clinical phenotype during variant prioritization. Their algorithms primarily emphasize variant-centric features (such as predicted functional impact, conservation, or population frequency) while the patient’s phenotype is only indirectly incorporated, typically through HPO terms. This imbalance can lead to the selection of variants with strong molecular features but limited clinical relevance, while overlooking variants that may better explain the phenotype. Consequently, clinical expertise remains essential to contextualize AI outputs and refine the prioritization process.
4. In IEI, as in other human diseases, the accuracy of phenotype-driven analyses is limited by the number and quality of available HPO terms. Clinical descriptions are often incomplete or overly general, restricting the ability of computational tools to match patient features with disease-associated genes. Enhancing the depth and standardization of HPO annotations (through more detailed clinical documentation and refined phenotype mapping) would greatly improve the capacity of AI-driven platforms to capture the clinical context and refine variant prioritization. It is worth noting that ongoing efforts are underway to expand and optimize HPO terms in IEI (Haimel et al., 2022; Maassen et al., 2023).
5. Current AI-driven platforms largely rely on curated databases such as ClinVar and OMIM, which may not always reflect the most recent discoveries. As a result, novel disease-gene associations or recently reported pathogenic variants can be overlooked. Integrating automated literature screening alongside database annotations could enhance the ability of these tools to identify potential genotype–phenotype correlations more comprehensively, improving variant prioritization and increasing diagnostic yield in challenging cases. This situation is well illustrated by the variant p. Gln101Ter in the *CD247* gene. The disease associated with *CD247* is typically autosomal recessive (AR), as reflected in OMIM (#610163). However, a recent study demonstrated that this variant can act through a dominant-negative mechanism, resulting in an autosomal dominant (AD) phenotype (Briones et al., 2024). At the time of this study, this information has not yet been updated in OMIM or other databases. Notably, one platform (AIM) still prioritized this variant, but linked it to the AR form of the disease.
6. Another important limitation of AI-driven platforms is their handling of VUS. By definition, VUS lack sufficient functional, population, or clinical evidence to be clearly classified as pathogenic or benign, which makes them inherently difficult for algorithmic prioritization. Moreover, as mentioned, these platforms often rely predominantly on variant-centric features (such as predicted molecular impact or conservation) while undervaluing the patient’s clinical phenotype. In our study, this dual challenge contributed to limited concordance between the VUS prioritized by AIM and AION, highlighting the continued need for expert manual curation to contextualize these variants in complex IEI cases.

In summary, based on our studies, AI-driven platforms represent a highly useful and time-efficient approach for the analysis of NGS data. For cases with clearly disease-causing variants, these tools can provide near-immediate results, significantly reducing the time and effort required for manual curation. In unsolved cases, there is still substantial potential to enhance AI-based variant prioritization, particularly by improving the integration of detailed patient phenotypes. Other limitations, such as reliance on curated databases, are more challenging to overcome. For example, databases predominantly contain functional evidence for coding variants, whereas non-coding variants (including those in regulatory elements, introns, or untranslated regions) are typically excluded due to limited functional annotation and clinical evidence. This constraint restricts the ability of current AI tools to detect potentially pathogenic variants outside the exome, including those affecting gene regulation or splicing. Future AI models would also benefit from moving beyond single-gene analyses, incorporating gene-gene interactions, pathway and protein-protein interaction data, and multi-omics information to provide a more comprehensive understanding of disease mechanisms. Ultimately, AI platforms capable of generating new hypotheses by critically integrating diverse data sources and proposing well-supported gene-disease relationships have the potential to transform rare disease research and clinical diagnostics.

## Data Availability

All relevant genetic information supporting the findings of this study is included within the manuscript and its Supplementary Materials. The complete whole-exome sequencing datasets of the patients have not been deposited in public repositories due to privacy and ethical restrictions. Additional details may be available from the corresponding authors upon reasonable request and in compliance with applicable regulations.
