# Supplementary material for: Exploring the strengths and limitations of AI-driven variant prioritization versus manual curation in inborn errors of immunity
Source: Front Genet. 2026 Mar 4;17:1713299. doi: 10.3389/fgene.2026.1713299 (PMC12995187; doi:10.3389/fgene.2026.1713299)
Supplement: Supplementary file 1 [file DataSheet1.pdf]

Supplementary Figure S1. Family trees of all included cases. Black shading indicates clinically affected individuals. Candidate variants classified as having *medium* or *high* clinical relevance are shown. All described variants are heterozygous, except for two variants indicated as homozygous (HOM).

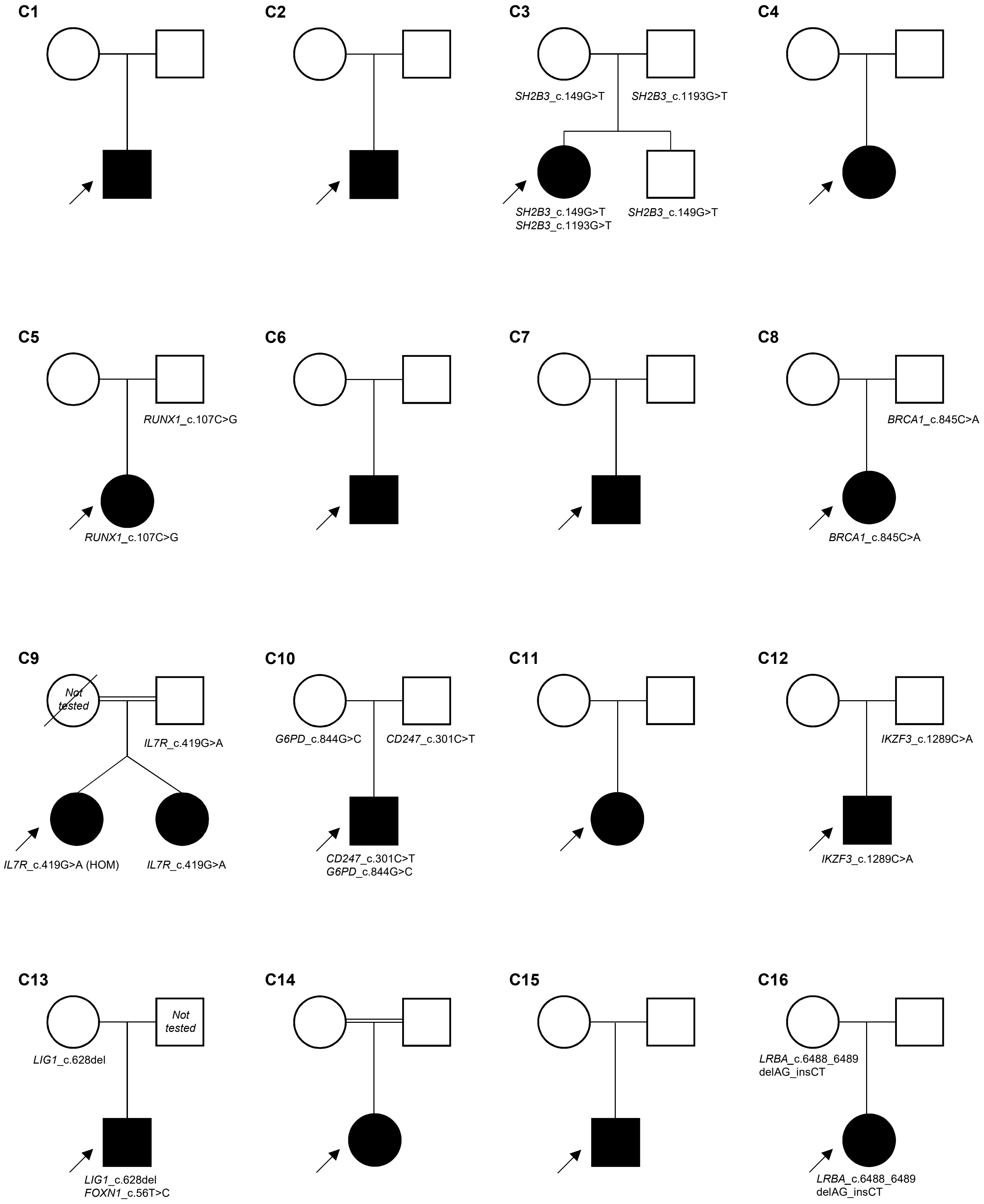

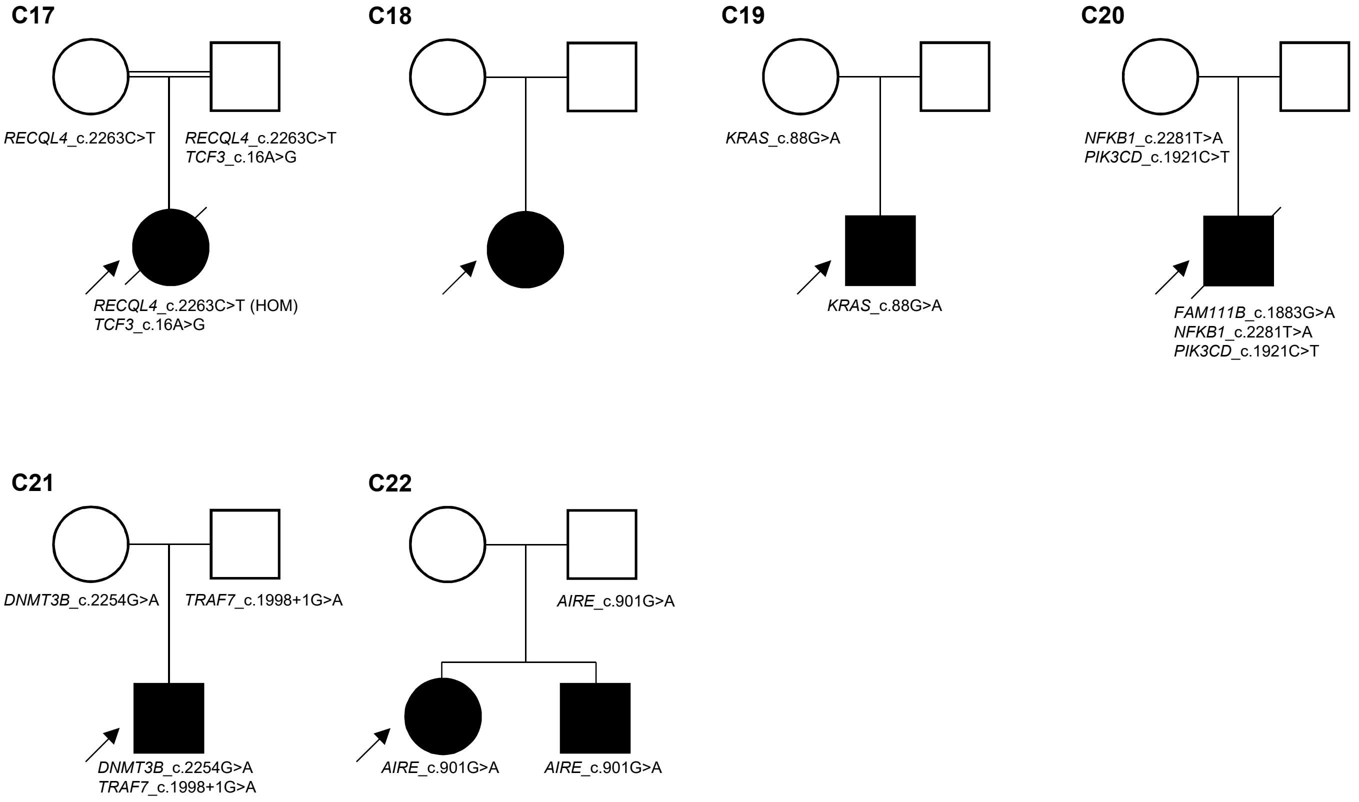
C17
RECQL4_c.2263C>T
RECQL4_c.2263C>T
TCF3_c.16A>G
RECQL4_c.2263C>T (HOM)
TCF3_c.16A>G
C18
C19
KRAS_c.88G>A
KRAS_c.88G>A
C20
NFKB1_c.2281T>A
PIK3CD_c.1921C>T
FAM111B_c.1883G>A
NFKB1_c.2281T>A
PIK3CD_c.1921C>T
C21
DNMT3B_c.2254G>A
TRAF7_c.1998+1G>A
DNMT3B_c.2254G>A
TRAF7_c.1998+1G>A
C22
AIRE_c.901G>A
AIRE_c.901G>A
AIRE_c.901G>A
